# Biomechanical analysis on custom-made insoles in gait of idiopathic pes cavus

**DOI:** 10.1186/1757-1146-7-S1-A131

**Published:** 2014-04-08

**Authors:** Jungkyu Choi, Ji Yong Jung, Yonggwan Won, Jung-Ja Kim

**Affiliations:** 1 Department of Healthcare Engineering, Chonbuk National University, Jeonju, Jeolabuk-do, 561-756, Korea; 2 School of Electronics and Computer Engineering, Chonnam National University, Gwangju, 500-757, Korea; 3 Division of Biomedical Engineering, Chonbuk National University, Jeonju, Jeolabuk-do, 561-756, Korea; 4 Research Center of Healthcare & Welfare Instrument for the Aged, Chonbuk National University, Jeonju, Jeolabuk-do, 561-756, Korea

## 

The purpose of this study was to evaluate the effects of custom-made insoles based on the foot pressures and electromyography (EMG) activities in a subject group of idiopathic pes cavus which the term used to describe a foot type with an excessively high medial longitudinal arch [1-3]. The study was conducted using on 10 persons who were diagnosed idiopathic pes cavus by a podiatrist (an age 22.3±0.08 years, a height 159.9±2.2 cm, a weight 50.8±3.69 kg, a foot size 237.9±3.27 mm, mean±SD) All subjects had no history of injury in the musculoskeletal system of the lower extremities except pes cavus. The subjects walked on a treadmill under two different experimental conditions: walking with Normal Shoes (NS) and walking with normal shoes equipped with custom-made insoles (CI) molded with the aim of reducing supination of pes cavus (Figure 1). When walking, plantar foot pressure data such as the maximum force (MF), the contacting area (CA), the peak pressure (PP) and the mean pressure (MP) were collected using Pedar-X System (Novel Gmbh, Germany) and EMG activity of lower limb muscles such as Rectus Femoris (RF), Tibialis Anterior (TA), Musculus Biceps Femoris (MBF) and Medial Gastrocnemius (MG) were also gathered using Delsys EMG Work System (Delsys, USA) [4-6]. Accumulated data was then analyzed using paired t-test in order to investigate the effects of each of experimental condition. As a result of the analysis, MF, PP and MP of midfoot were increased by increased CA of midfoot on CI condition, so CA and MF of forefoot and rearfoot were decreased. In addition, PP and MP of rearfoot were decreased significantly. As a result of the analysis in the view point of stance phase, MF, PP and MP in the initial contact and the loading response were decreased significantly on CI condition. In the mid stance, MF, PP and MP were increased significantly by the increased CA of midfoot on CI condition. MF and PP were also increased significantly in the terminal stance but MP was decreased (Table 1). In case of EMG, all the muscle activities were decreased significantly on CI condition. An important contribution of this study is an analysis of all the changes in a muscle activities caused by wearing the custom-made insoles. Thus, the result of this study can be applied for designing functional insoles and lower extremity orthoses for individuals with pes cavus.

**Figure 1:**
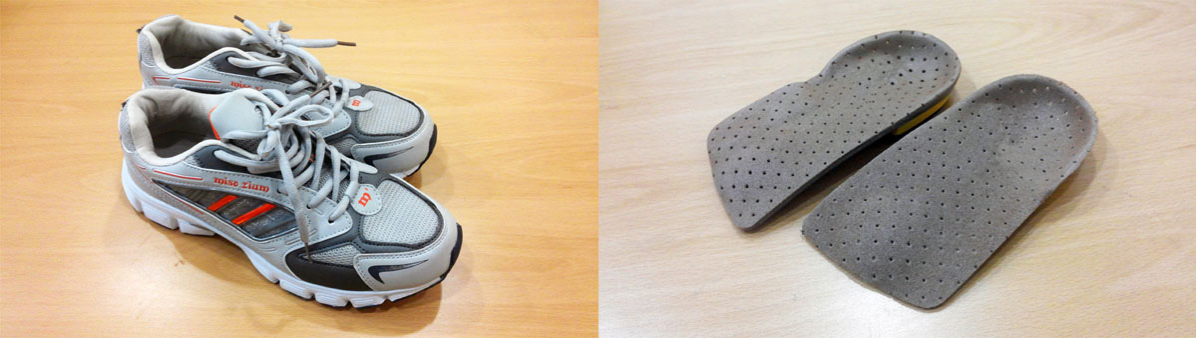
Normal Shoes (NS) and Custom-made Insoles (CI)

**Table 1:** The results of plantar pressure in stance phase.

		Normal shoes (NS)		Custom-made Insoles (CI)	
		
		mean	SD	mean	SD
Contacting area (cm^2^)	Initial contact	15.82	9.94	17.43	10.78
	
	Loading response	42.25	17.92	40.97	13.7
	
	Mid stance	74.18	4.21	74.9	5.79
	
	Terminal stance	47.17	14.32	57.03*	15.25

Maximum force (N)	Initial contact	55.65	44.15	53.66	44.65
	
	Loading response	254.15	125.91	243.16*	120.7
	
	Mid stance	400.55	11.06	425.76*	38.87
	
	Terminal stance	283.42	128.78	335.14*	156.3

Peak pressure (kPa)	Initial contact	41.5	28.26	36.75*	28.71
	
	Loading response	115.07	41.63	112.14*	36.38
	
	Mid stance	127.05	17.04	139.61*	19.58
	
	Terminal stance	128.97	45.55	143.57*	41.37

Mean pressure (kPa)	Initial contact	25.51	16.58	22.75*	13.69
	
	Loading response	55.42	16.72	54.27	16.15
	
	Mid stance	54.84	4.83	57.71*	5.01
	
	Terminal stance	56.45	15.02	54.83*	15.24

* p < 0.05 significant difference between NS and CI

## Trial registration

Current Controlled Trials ISCRTN73824458
